# Bedside Ultrasound Measurement of Rectus Femoris: A Tutorial for the Nutrition Support Clinician

**DOI:** 10.1155/2017/2767232

**Published:** 2017-03-13

**Authors:** Carlos Alfredo Galindo Martín, Enrique Monares Zepeda, Octavio Augusto Lescas Méndez

**Affiliations:** ^1^Nutrition Department, Hospital San Ángel Inn Universidad, Ciudad de México, Mexico; ^2^Intensive Care Unit, Hospital San Ángel Inn Universidad, Ciudad de México, Mexico

## Abstract

Intensive care unit acquired weakness is a long-term consequence after critical illness; it has been related to muscle atrophy and can be considered as one of the main nutritional support challenges at the intensive care unit. Measuring muscle mass by image techniques has become a new area of research for the nutritional support field, extending our knowledge about muscle wasting and the impact of nutritional approaches in the critical care setting, although currently there is no universally accepted technique to perform muscle measurements by ultrasound. Because of this, we present this tutorial for nutrition support clinicians, in order to understand and perform muscle measurements by this reliable, accessible, low-cost, and easy-to-use technique. Reviewing issues such as quadriceps muscle anatomy, correct technique (do's and don'ts), identification of structures, and measurement of the rectus femoris and vastus intermedius muscles helps to acquire the basic concepts of this technique and encouraging more research in this field.

## 1. Background

It has been observed that most of the critically ill patients who survive acute respiratory distress syndrome (ARDS) have to deal with a wide variety of consequences, including muscular wasting and weakness, and these conditions could last for at least one year [1].

Intensive care unit acquired weakness (ICUAW) has been defined as generalized weakness that develops during critical illness and where no other explanation than critical illness is present [2] and is associated with long-term consequences from the medical, human, and socioeconomic point of view [3].

Muscular atrophy has been proposed as the universal feature in patients with ICUAW and it can start in early stages of critical illness (within hours of onset of the disease) and its development has been related to the acute inflammatory process and immobilization. Factors as age, muscular function prior to critical illness, medications, comorbidities, nutrition, nervous, and muscular damage can contribute positively to the extent of the damage and negatively in muscular and functional recovery capacity [4].

From the nutrition perspective, one of the main challenges of providing nutritional support to critically ill patients is to stop or slow lean mass losses [5]. For these reasons, it is fundamental for the nutritional support clinician to be able to measure and assess muscle wasting during critical illness, using an easy and accessible technique.

Body composition measurements have a fundamental value in the comprehensive nutritional assessment. There are multiple methods to conduct these measurements, for example, anthropometry, corporal density/volume, hydrometry, mayor elements, bioimpedance, and image techniques [6].

In the case of the classic anthropometric measurements such as circumferences, its validity is affected by fluid overload, a common situation in the critically ill patient [7].

Other techniques such as hydrodensitometry, plethysmography, dilution techniques, total body count, neutron activation analysis, dual X-ray absorptiometry (DEXA), computed tomography, and magnetic resonance are difficult to perform due to multiple causes, such as the need of specialized personnel, high costs, and difficulties in patient transport. reducing their applicability in the critical care setting [6].

It has been shown that muscle mass measurement by ultrasonography is a reliable technique in most of the patients even when edema and fluid retention are present [8]. Recently it has been tested in patients with acute kidney injury and renal replacement therapy, showing good intra- and interobserver correlations and no significant chances in measurement before and after the renal replacement therapy [9]. Muscle mass loss in critically ill patients has been assessed by US, histological, and molecular biology techniques, showing a significant reduction of approximately 10% of the rectus femoris (RF) cross-sectional area (CSA) measured by US correlating with a decrease in muscle fibers CSA and less protein synthesis [10]. Also myofiber necrosis and muscular fascia inflammation have been found [11].

US has become a widely used research technique to quantify muscle wasting showing remarkable accuracy and reliability [12] with strong clinimetric properties [13] and excellent intra- and interobserver reliability in healthy people measured by clinicians with no previous experience in US [14].

For these reasons, we designed this tutorial in order to guide the nutrition support clinician without experience in US, to perform muscle measurements, principally RF and vastus intermedius (VI), being able to apply these techniques in further research in the critical care (real) setting, improving the knowledge about muscle wasting and its correlation with nutritional support.

For the scope of this article, we are focusing on different techniques used for their practicality, supported by previous literature and anatomical and functional principles. Basic concepts of ultrasonography can be reviewed elsewhere [15, 16].

## 2. Description of the Technique

### 2.1. What?

Lower limbs muscles are prone to early atrophy, showed by a greater decrease of thickness within the first five days of admission to the intensive care unit compared with upper limbs, making these muscles a good target for muscle mass assessment [17].

### 2.2. Where?

The quadriceps femoris is a group of muscles composed by three vastus muscles (medialis, intermedius, and lateralis) and the RF. The latter one presents a proximal insertion in the anterior inferior iliac spine (AIIS) and other insertion in the supraacetabular sulcus. The quadriceps femoris is distally inserted in the tibial tuberosity by a common ligament and is a hip flexor and a knee extensor [18, 19].

Before starting, make sure the patient is in supine position with extended knees and toes pointing to the ceiling. This is the most used position in this kind of measurements [12]. This position helps the practitioner to place the patient in the same static position every time; using an angle (ex. 30° or 45°) of head of bed elevation could introduce some error when performing the measurement time to time.

**Figure 1:**
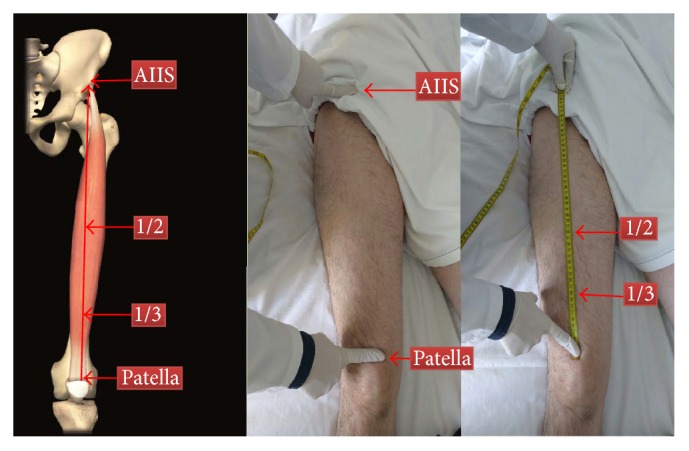
Finding the right place to measure. AIIS: anterior inferior iliac spine.

Multiple landmarks have been used; although there is currently no consensus or universally accepted landmark, an accessible landmark should be used. Given that the patient is usually face up, we propose the following technique: using a nonstretchable measuring tape, trace an imaginary line in the anterior part of the thigh from the AIIS to the midpoint of the proximal border of the patella and mark the middle and one-third point between these two references which easily give us access to the RF and VI (Figure 1). The reason to use de AIIS and not the anterior superior iliac spine is because using the exact middle point of the muscle helps us to find its thickest part using as reference the insertion points of this muscle (RF) and the reason to use a third of the distance will be discussed latter.

We recommend performing the measurement in the direction from the patella to the AIIS, since this final point is hard to find in some populations, principally obese patients (Principal Arrow in Figure 1).

It seems practical to use a permanent marker and this way we ensure that the measurement is made at the same point each time [8].

### 2.3. How?

US images are a real time and thomographic view of anatomical structures [19] obtaining a longitudinal or transversal (cross-sectional) image [16].

For muscle mass assessment, US equipment with bidimentional mode is required, and also a linear transducer or probe (frequency: 7–13 MHz) which allow us to obtain high resolution images of superficial structures [12, 16]. A higher frequency generates higher resolution at the expense of reduced image depth [20].

*Note*. Before starting, the measurement is important to assess the eligibility of each patient, given that those patients with fractures, lesions, or burns in the section of interest should not be included in the protocol. It should be discussed if patients with neuromuscular diseases also are not eligible for this technique.

**Figure 2:**
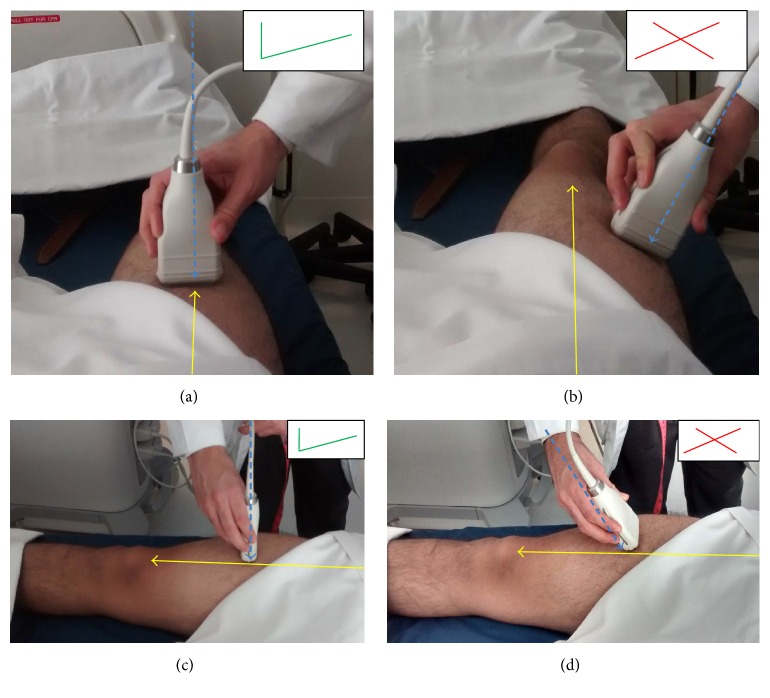
Transducer do's and don'ts. Anterior positioning of the transducer: (a) correct (do) and (b) incorrect (don't). Transducer angle: (c) correct (do) and (d) incorrect (don't).

To obtain a cross-sectional image, the transducer must be oriented transversally to the longitudinal axis (the imaginary line marked before) of the thigh forming a 90° angle in relation to the skin surface (Figure 2). Tilting or moving the probe from its original position and angle will contribute to obtaining an incorrect measurement.

Once the image has been obtained, the following structures must be identified (Table 1).

*Note*. Adjust the depth of the image to find the femur using the “depth” control.

It is important to mention that, depending on the type of measurement, we want to take the position and the pressure of the probe will have to vary. Both techniques with minimum and maximum pressure have shown good intra- and interobserver correlations in critically ill patients [14, 21, 22]. Even so, if it is required to obtain the CSA of the RF, the proper point is one-third using minimal pressure so that the entire muscle is visible. For the measurement of thicknesses, both RF and VI together or both landmarks separated using minimal pressure seem useful. The techniques that use maximum pressure are more adequate to only measure the thickness of both muscles together.

**Figure 3:**
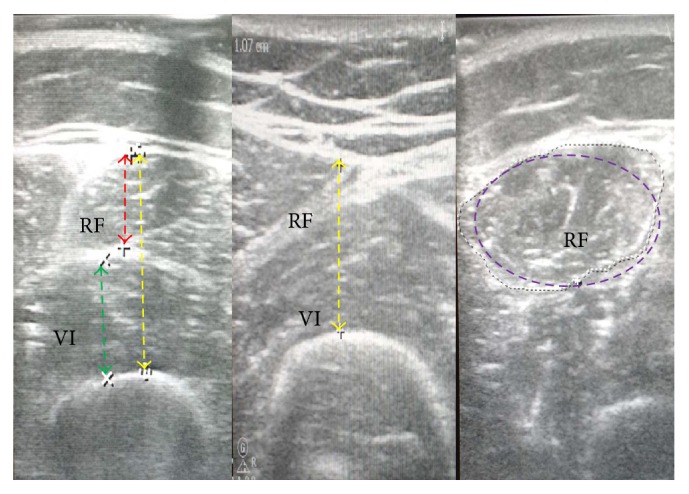
Measurements. RF: rectus femoris; VI: vastus intermedius. From left to right: thicknesses using minimal pressure, thickness using maximal pressure, and cross-sectional area (landmark: 1/3 using minimal pressure).

For image acquisition, use the “Freeze” button to obtain a static image. Subsequently using planimetric techniques to measure the distances and areas: (1) thickness: from the fat-muscle interface to the muscle-muscle interface or from the fat-muscle interface to the bony surface [12] forming a horizontal straight line and (2) outlining manually the area of the RF (Figure 3).

*Note*. Repeat this measurement 3 times and use the average as the final value.

## 3. Conclusion

At the present time, we “know” that critically ill patients develop undernutrition during their ICU stay, but, until we know how to assess this condition in real time and bedside, all nutritional approaches would be directed to cure or reverse undernutrition; the perfect scenario would be to prevent it. This new technique holds great promises in the future given its bedside applicability, but it is imperative to follow a standardized protocol to reduce variations and to document changes in measurements performed at different times [23].

## Figures and Tables

**Table 1:** Identification of structures.

Order	Structure	Description
1	Skin	Hyperechoic layer adjacent to the transducer [18].
2	Subcutaneous tissue (fat)	Hypoechoic layer of variable thickness with hyperechoic lines resembling a feather [18].
3	Muscular fascia	Hyperechoic layer corresponding to the first interface where the RF interposes.
4	Rectus femoris	Semicircle structure delimited by the muscular fascia and the second interface.
5	Second interface	Hyperechoic layer where the VI interposes.
6	Vastus intermedius	Rectangular structure delimited by the second interface and the bony surface.
7	Bony surface	Hypoechoic circular structure delimited (acoustic shadow) by a hyperechoic layer corresponding the femur cortical layer (sonic surface) [18].

## Abbreviations

ICUAW: Intensive care unit acquired weakness
DEXA: Dual X-ray absorptiometry
RF: Rectus femoris
CSA: Cross-sectional area
VI: Vastus intermedius
AIIS: Anterior inferior iliac spine.
